# iPseU-NCP: Identifying RNA pseudouridine sites using random forest and NCP-encoded features

**DOI:** 10.1186/s12864-019-6357-y

**Published:** 2019-12-30

**Authors:** Thanh-Hoang Nguyen-Vo, Quang H. Nguyen, Trang T.T. Do, Thien-Ngan Nguyen, Susanto Rahardja, Binh P. Nguyen

**Affiliations:** 1 School of Mathematics and Statistics, Victoria University of Wellington, Gate 7, Kelburn Parade, Wellington, 6140 New Zealand; 2 School of Information and Communication Technology, Hanoi University of Science and Technology, 1 Dai Co Viet, Hanoi, 100000 Vietnam; 3 Institute of Research and Development, Duy Tan University, Danang, 550000 Vietnam; 4 Computational Biology Center, International University - VNU HCMC, Quarter 6, Linh Trung Ward, Thu Duc District, Ho Chi Minh City, 700000 Vietnam; 5 School of Marine Science and Technology, Northwestern Polytechnical University, 127 West Youyi Road, Xi’an, 710072 China

**Keywords:** RNA, Pseudouridine site, Uridine, Identification, NCP, Random forest

## Abstract

**Background:**

Pseudouridine modification is most commonly found among various kinds of RNA modification occurred in both prokaryotes and eukaryotes. This biochemical event has been proved to occur in multiple types of RNAs, including rRNA, mRNA, tRNA, and nuclear/nucleolar RNA. Hence, gaining a holistic understanding of pseudouridine modification can contribute to the development of drug discovery and gene therapies. Although some laboratory techniques have come up with moderately good outcomes in pseudouridine identification, they are costly and required skilled work experience. We propose iPseU-NCP – an efficient computational framework to predict pseudouridine sites using the Random Forest (RF) algorithm combined with nucleotide chemical properties (NCP) generated from RNA sequences. The benchmark dataset collected from Chen et al. (2016) was used to develop iPseU-NCP and fairly compare its performances with other methods.

**Results:**

Under the same experimental settings, comparing with three state-of-the-art methods including iPseU-CNN, PseUI, and iRNA-PseU, the Matthew’s correlation coefficient (MCC) of our model increased by about 20.0%, 55.0%, and 109.0% when tested on the *H. sapiens* (H_200) dataset and by about 6.5%, 35.0%, and 150.0% when tested on the *S. cerevisiae* (S_200) dataset, respectively. This significant growth in MCC is very important since it ensures the stability and performance of our model. With those two independent test datasets, our model also presented higher accuracy with a success rate boosted by 7.0%, 13.0%, and 20.0% and 2.0%, 9.5%, and 25.0% when compared to iPseU-CNN, PseUI, and iRNA-PseU, respectively. For majority of other evaluation metrics, iPseU-NCP demonstrated superior performance as well.

**Conclusions:**

iPseU-NCP combining the RF and NPC-encoded features showed better performances than other existing state-of-the-art methods in the identification of pseudouridine sites. This also shows an optimistic view in addressing biological issues related to human diseases.

## Background

Two decades have seen a significant growth in ‘omics’ science - a multidisciplinary scientific field combining base knowledge of molecular genetics, advanced experimental techniques, and availability of powerful computing sources as well as novel computational frameworks to solve many different biological issues. In recent years, the explosion of ‘omics’ data has further provided scientists valuable sources to explore molecular behaviors of various biochemical pathways with hopes in seeking better solutions to human illnesses. ‘Omics’ science covers four main subfields, including genomics, transcriptomics, proteomics, and metabolomics, which are corresponding to four expression levels. In this study, we focus on identifying RNA Pseudouridine sites (RPS) which is one the hot topic in transcriptomics. Pseudouridine (*Ψ*) has been known as one of the most essential RNA modifications found in both prokaryotes and eukaryotes [1], and this biochemical event randomly and unexpectedly occurs in any types of RNA [2]. During the reaction, *Ψ* synthase enzyme cleaves a uridine residue from its original nucleoside to add a *Ψ* residue, an isomer of uridine, by rotating a bonding angle along the N3–C6 axis at 180^∘^ and finally form a new bond between the base’s 5-carbon and the 0-carbon of the nucleoside. Recent studies have claimed the vital role of this biochemical event in transcriptional activities due to its contribution in maintaining the functional structure of tRNA [3, 4] and gene regulation machine (e.g., spliceosome). Besides, *Ψ* modification can accelerate RNA-RNA/RNA-protein interaction and spliceosome assembling [5]. Furthermore, *Ψ*-incorporated mRNAs can restrict the RNA-recalled innate immune response and intensify the activity of mRNAs during the translation [6]. Despite being under investigations for more than half of the century, neither biological functions nor enzymatic mechanisms of Pseudouridine have been fully explored. Hence, looking for new methods to identify RPS may come up with answers for many undisclosed biological mysteries.

For years, scientists have introduced different laboratory techniques to identify RPS but they are costly and required skilled work experience [7–9]. Therefore, developing advanced and low-cost methods that can simplify original work is necessary. Recently, the explosion of ‘omics’ data provides huge valuable sources for knowledge discovery targeting various biological issues via faster, more powerful, and more affordable strategies taking advantages of computational advances. Several in silico studies have been conducted to identify RPS using machine learning algorithms [10–12]. In 2015, Li et al. introduced PPUS [13] – a prediction framework combining the Support Vector Machines (SVM) and features extracted from surrounding nucleotides to detect *Ψ* synthase (PUS)-specific *Ψ* sites (of *S. cerevisiae* and *H. sapiens*). In 2016, iRNA-PseU, another SVM framework using the modified platform of pseudo-k-tuple nucleotide composition (PseKNC), was proposed by Chen et al. [14]. The development of iRNA-PseU used a benchmark dataset of *M. musculus*, *S. cerevisiae*, and *H. sapiens*. Two years later, He et al. developed a different SVM model called PseUI [15] adopting selected features from five encoding schemes including position-specific dinucleotide propensity (PSDP), position-specific nucleotide propensity (PSNP), pseudo dinucleotide composition (PseDNC), nucleotide composition (NC), and dinucleotide composition (DC). Most recently, Tahir et al. introduced iPseU-CNN and demonstrated that using deep neural networks can improve performances in identifying RPS [16]. Although these models have provided good performance, the hunt for models of better performance and adequate complexity in biomedical field is an ongoing research that is always imperative.

In this study, we introduce iPseu-NCP, a simpler but better computational framework, for identifying RPS. iPseu-NCP is developed using the Random Forest (RF) and nucleotide chemical properties (NCP) feature. The combination between a powerful tree-based ensemble learning algorithm and a simple but effective encoding scheme massively accelerates computing speed as well as reduces model complexity. To fairly assess the model performance between iPseu-NCP and other methods, the benchmark dataset introduced in Chen et al.’s study [14] was used for model development and evaluation.

## Results and Discussions

### Sequence Analysis

To compare the biological patterns among the three development sets (*H. sapiens*, *S. cerevisiae*, and *M. musculus*), sequence logo visualization using Two Sample Logo with independent *t*-test (*p*<0.05) [17] was used. A first idea of displaying consensus sequences started from Schneider et al. [18] when they wanted to visualize common biologcal patterns in a set of aligned sequences. Each sequence-logo plot carries information about (a) the most frequent nucleotides counting from the top of each particular position, (b) the occurrence frequency of each nucleotide indicated by the proportional height of the letter, and (c) the significance of each particular position adjusted by height of the whole stack of letters.

**Fig. 1:**
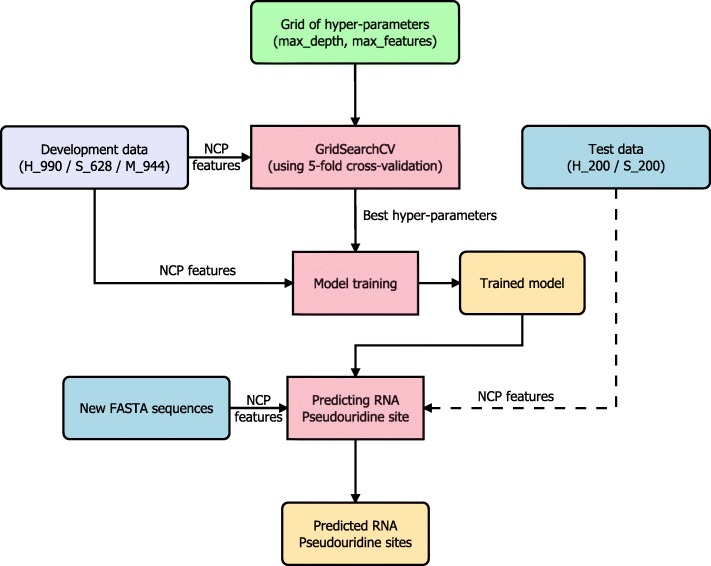
Sequence characteristics of *ψ* sites across the three species, including (**a**) *H. sapiens*, **b***S. cerevisiae*, and **c***M. musculus*. Sequence analysis using logo representations were created by Two Sample Logo with *t*-test (*p*<0.05)

For each development set in this study, a significance testing for the difference between positive sequences (or *Ψ*-site holders) and the negative sequences (or non- *Ψ*-site holders) was performed. The plot gives information about two groups of nucleotides found in the positive set with the negative set is used as a base for comparison. A nucleotide which is frequently found in a particular position of many positive samples is termed as ‘enriched nucleotide’. A nucleotide which is rarely found in a particular position of many positive samples is termed as ‘depleted nucleotide’. Based on the occurrence frequencies a nucleotide at a particular position, *t*-test was performed to find whether the certain occurrence of a nucleotide is random or directional assuming that four types of nucleotide randomly appear and their distributions in the positive set and negative set are identical. It can be observed that guanine (G) in *H. sapiens* and *S. cerevisiae* are significantly enriched at multiple positions with 17.6% and 22.9%, respectively. For *H. sapiens*, uracil (U) and adenine (A) are two major depleted nucleotides while for *S. cerevisiae*, these occurrence frequencies seem to be equally shared by uracil (U), adenine (A), and cytosine (C). In terms of *M. musculus*, adenine (A) is the first enriched nucleotide at the position right next to the *Ψ* site (position numbered 12), followed by cytosine (C) at the position numbered 13, and uracil (U) at the position numbered 9, 12, and 13. On the other hand, regarding this species, adenine (A) is the main significantly depleted nucleotide at multiple positions. At the position numbered 13 of sequences in *H. sapiens* and *M. musculus*, a same biological pattern can be observed and this fact somehow may indicate close evolutionary distance between *H. sapiens* and *M. musculus* compared to *H. sapiens* - *S. cerevisiae* pair as well as *M. musculus* - *S. cerevisiae* pair (Fig. 1).

### Comparative screening on Encoding Schemes

**Fig. 2:**
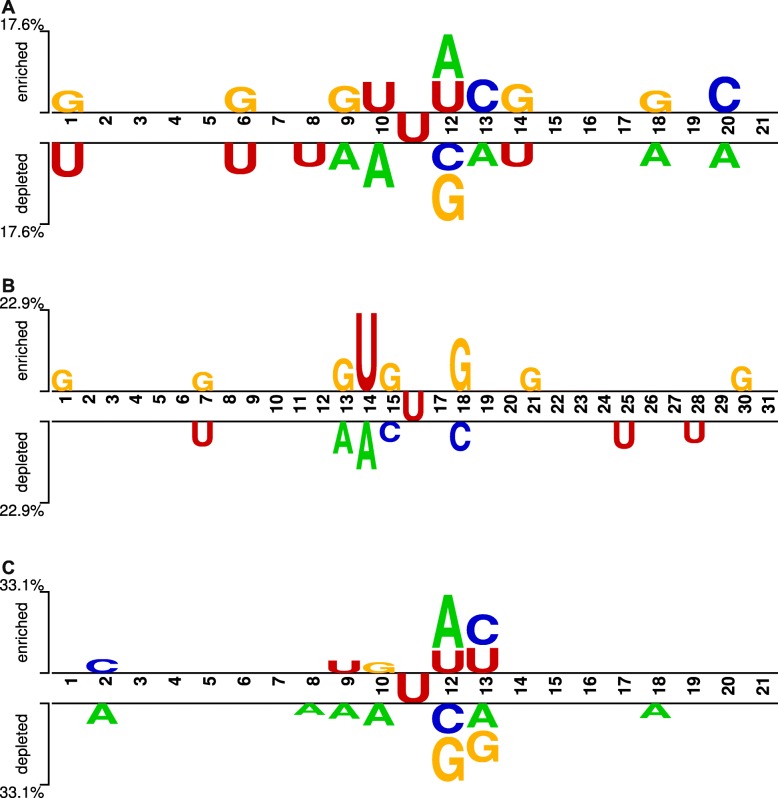
The processing steps in the proposed framework

Although NCP is a relatively simple encoding scheme, its uniqueness comes from the specific chemical nature of each type of ribonucleic acid. Based on three chemical properties, there is no ribonucleic acid sharing more than one property with others. Therefore, NCP-encoded features extracted from an RNA sequence contain sufficient structural information for a binary classification problem. For a comparison, two other encoding schemes including the pseudo-k-tuple nucleotide composition (PseKNC) [19] and the composition of k-spaced nucleic acid pairs (CKSNAP) [20] were also tested with our RF models in such the same way as NCP (Fig. 2). These two encoding schemes have been used in a number of research works, especially, PseKNC was the encoding scheme in both iRNA-PseU and PseUI. The 5-fold cross-validation results in Table 1 show that NCP outperformed the other two encoding schemes in all the three development datasets and in almost all the evaluation metrics, especially accuracy and MCC. This confirms the effectiveness of NCP when using with the RF classifier in identifying RPS.

**Table 1:** Comparative analysis on our RF model using different encoding schemes under 5-fold cross-validation on different development datasets

Dataset	Encoding Scheme	ACC (%)	SN (%)	SP(%)	MCC
**H_990**	PseKNC	59.39	69.49	49.29	0.19
	CKSNAP	60.00	**83.84**	36.16	0.23
	NCP	**62.92**	58.79	**65.05**	**0.24**
**S_628**	PseKNC	58.76	51.91	**65.61**	0.18
	CKSNAP	60.03	56.37	63.69	0.20
	NCP	**69.59**	**77.07**	62.10	**0.40**
**M_944**	PseKNC	56.57	44.49	68.64	0.14
	CKSNAP	57.52	52.54	62.50	0.15
	NCP	**71.82**	**67.37**	**76.27**	**0.44**

Values which are significantly higher than the others are in bold

### Feature Importances

**Fig. 3:**
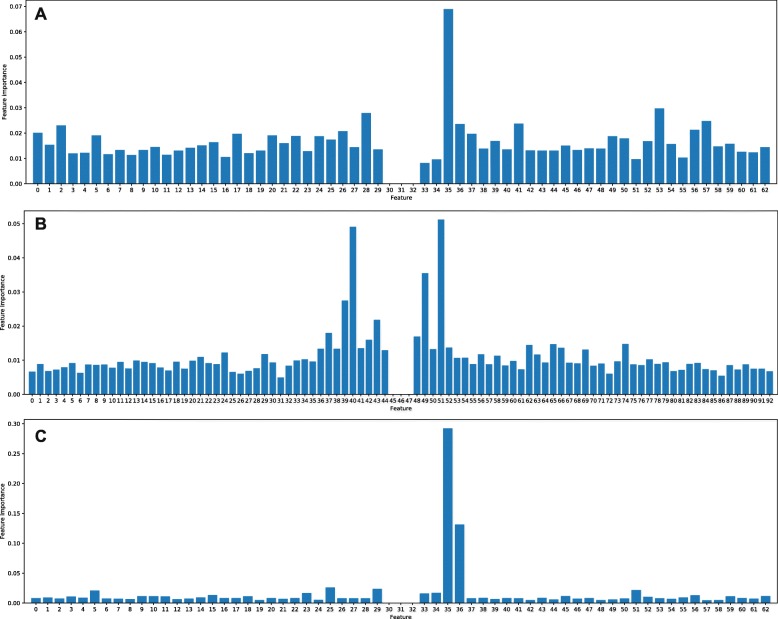
Feature importances of the three predictive models for (**a**) *H. sapiens*, **b***S. cerevisiae*, and **c***M. musculus*

Figure 3 presents the feature importance ranking for the RF models. Since each sequence in H, M, and S development set has exactly 21, 21, and 31 nucleotides, respectively. The number of generated NCP features are 3×*n* = 63, 63, and 93, respectively, where *n* is the sample length. The three central features of each sample (features numbered 30, 31, and 32 for H and M set, and features numbered 45, 46, and 47 for S set) are zero-important because the corresponding nucleotide is always ‘U’ leading to a non-specificity in distinguishing these samples from each other. For H set, feature numbered 35 belonging to the corresponding nucleotide numbered 17 is ranked dominantly higher than other features. Some features such as 2, 28, 41, 53, and 57 are also slightly higher than the rest of the features but not significant as well as disorderedly distributed. With regard of S set, features numbered from 36 to 44 and from 48 to 52 which are corresponding to nucleotides numbered 13, 14, 15, 17, and 18 are ranked as important features compared to the others. The importance of these other features leaps down to the two ends of the sequence. For M set, there is a completely reverse trend compared to the other sets with most of the features (higher than 95%) being considered as distinguishingly non-essential. Features numbered 35 and 36 of the corresponding nucleotides numbered 17 and 18 are ordered as far more important than the others. From the data distribution of the generated features, the importance of near nucleotides surrounding the central ‘U’ is confirmed with clear evidence and the importance of nucleotides decreases when their distances from the central ‘U’ increase.

### Cross-Validation and Model Evaluation

**Fig. 4:**
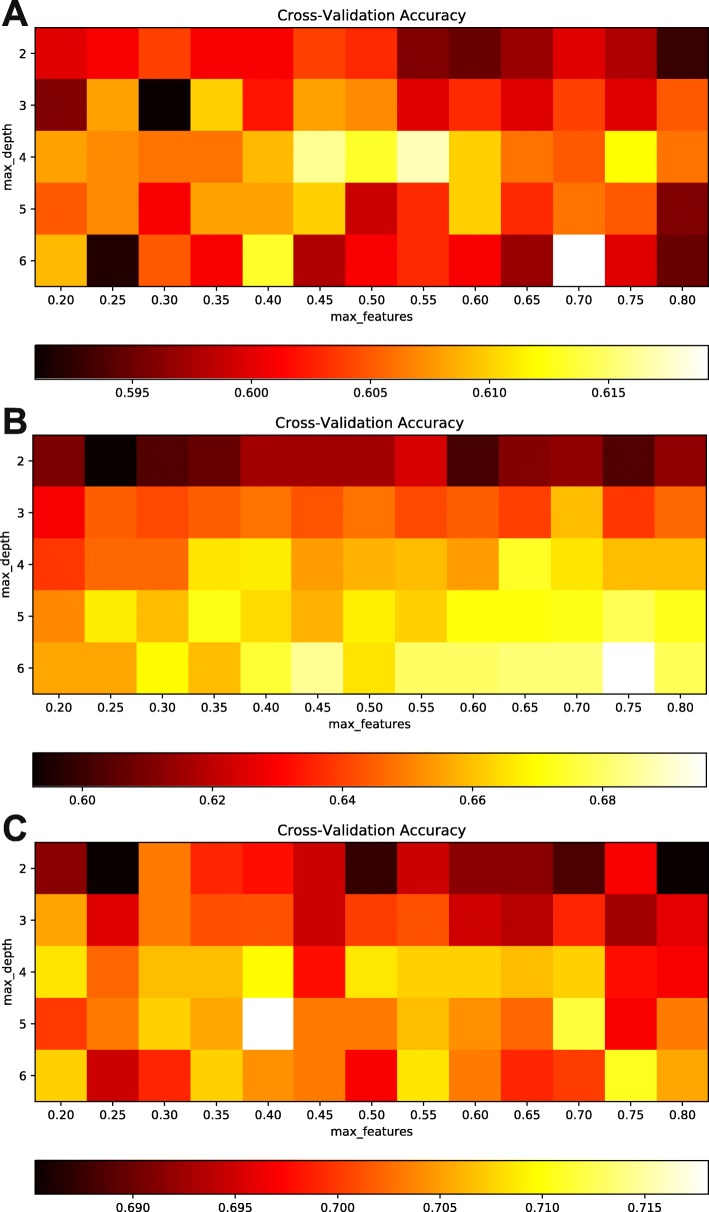
Heatmap indicating the 5-fold cross-validation accuracy with different combinations of max_depth and max_features across the three development datasets, including **a** H_990, **b** S_628, and **c** M_944

Figure 4 describes the process of finding optimal values for two hyper-parameters max_depth and max_features. For dataset H, max_depth of 6 and max_features of 0.70 came up with better accuracy compared to the others, while for datasets S and M, the hyper-parameter pair (max_depth, max_features) are (6, 0.75) and (5, 0.40), respectively. In comparison between our method and the state-of-the-art methods using 5-fold cross-validation, significant improvement in model performance was noticed besides some limitations that need to be addressed. For dataset H, iPseU-CNN of Tahir et al. remains higher than our methods as well as the other previous ones. For dataset S, except specificity, our method has come up with remarkable results compared to the others while for dataset M, our method and iPseU-CNN share equal values of accuracy and MCC besides the considerable increase in specificity (Table 2).

**Table 2:** Comparative analysis between results of the proposed method and other studies using 5-fold cross-validation

Dataset	Model	ACC (%)	SN (%)	SP (%)	MCC	Method
**H_990**	iRNA-PseU	60.40	61.01	59.80	0.21	Chen et al., 2016
	PseUI	64.24	64.85	63.64	0.28	He et al., 2018
	iPseU-CNN	**66.68**	**65.00**	**68.78**	**0.34**	Tahir et al., 2019
	iPseU-NCP	62.92	58.79	65.05	0.24	**Ours**
**S_628**	iRNA-PseU	64.49	64.65	64.33	0.29	Chen et al., 2016
	PseUI	65.13	62.74	67.52	0.30	He et al., 2018
	iPseU-CNN	68.15	66.36	**70.45**	0.37	Tahir et al., 2019
	iPseU-NCP	**69.59**	**77.07**	62.10	**0.40**	**Ours**
**M_944**	iRNA-PseU	69.07	73.31	64.83	0.38	Chen et al., 2016
	PseUI	70.44	**79.87**	70.34	0.41	He et al., 2018
	iPseU-CNN	71.81	74.79	69.11	0.44	Tahir et al., 2019
	iPseU-NCP	**71.82**	67.37	**76.27**	**0.44**	**Ours**

Values which are significantly higher than the others are in bold. Data excerpted from [16]

### Comparative Analysis on Independent Datasets

To fairly assess the model performance, we compare our method with existing state-of-the-art methods. Since the independent datasets (H_200 and S_200) have only sequence data from *H. sapiens* and *S. cerevisiae*, comparison among these methods was not taken account of *M. musculus*. For H_200, our iPseU-NCP model produced a significant improvement in accuracy, specificity, and MCC by about 7.0%, 30.0%, and 20.0% compared to those of iPseU-CNN - the current best method developed by Tahir et al. while the sensitivity of our model is only about 10.0% lower than that of iPseU-CNN. For S_200, our results indicate a considerable performance growth in accuracy, sensitivity, and MCC by about 2.0%, 6.0%, and 6.5% compared to those of iPseU-CNN. In fact, although the specificity of iPseU-NCP is numerically lower than iPseU-CNN, this difference is absolutely ignorable because it is not statistically different.

According to the experimental results, significant growths in MCC for the two independent test sets show that iPseU-NCP remarkably improved the model stability and performance compared to other previous methods. For H_200, in comparison with iPseU-CNN, PseUI, and iRNA-PseU, the MCC of iPseU-NCP considerably increased by about 20.0%, 55.0%, and 109.0%, respectively. For S_200, the MCC of iPseU-NCP intensively increased by about 6.5%, 35.0%, and 150.0% when compared to iPseU-CNN, PseUI, and iRNA-PseU, respectively. This improvement is highly meaningful in model construction to ensure the reliability in binary classification problems [21]. Besides, MCC is supposed to be more informative than accuracy because it considers the proportion of all the four components (TF, TN, FP, and FN) of the confusion matrix [21].

On the other hand, in terms of the accuracy when tested on the two independent test sets (H_200 and S_200), iPseU-NCP also archived better performance with a success rate boosted by 7.0%, 13.0%, and 20.0% and 2.0%, 9.5%, and 25.0% compared to iPseU-CNN, PseUI, and iRNA-PseU, respectively. Testing on H_200 also resulted in the improved specificity of iPseu-NCP by about 28.0%, 15.0%, and 20.0% compared to iPseU-CNN, PseUI, and iRNA-PseU, respectively while testing on S_200, the sensitivity of iPseu-NCP raised by about 7.0%, 12.0%, and 16.0% compared to iPseU-CNN, PseUI, and iRNA-PseU, respectively (Table 3). Briefly, the essential growth in both accuracy and MCC for dataset H indicates better model fitness to address the molecular genetics issues related to human beings.

**Table 3:** Comparative analysis between results of the proposed method and other studies on the independent test sets

Dataset	Model	ACC (%)	SN (%)	SP (%)	MCC	Method
**H_200**	iRNA-PseU	61.50	58.00	65.00	0.23	Chen et al., 2016
	PseUI	65.50	63.00	68.00	0.31	He et al., 2018
	iPseU-CNN	69.00	**77.72**	60.81	0.40	Tahir et al., 2019
	iPseU-NCP	**74.00**	70.00	**78.00**	**0.48**	**Ours**
**S_200**	iRNA-PseU	60.00	63.00	57.00	0.20	Chen et al., 2016
	PseUI	68.50	65.00	72.00	0.37	He et al., 2018
	iPseU-CNN	73.50	68.76	**77.82**	0.47	Tahir et al., 2019
	iPseU-NCP	**75.00**	**73.00**	77.00	**0.50**	**Ours**

Values which are significantly higher than the others are in bold. Data excerpted from [16]

### Software Availability

**Fig. 5:**
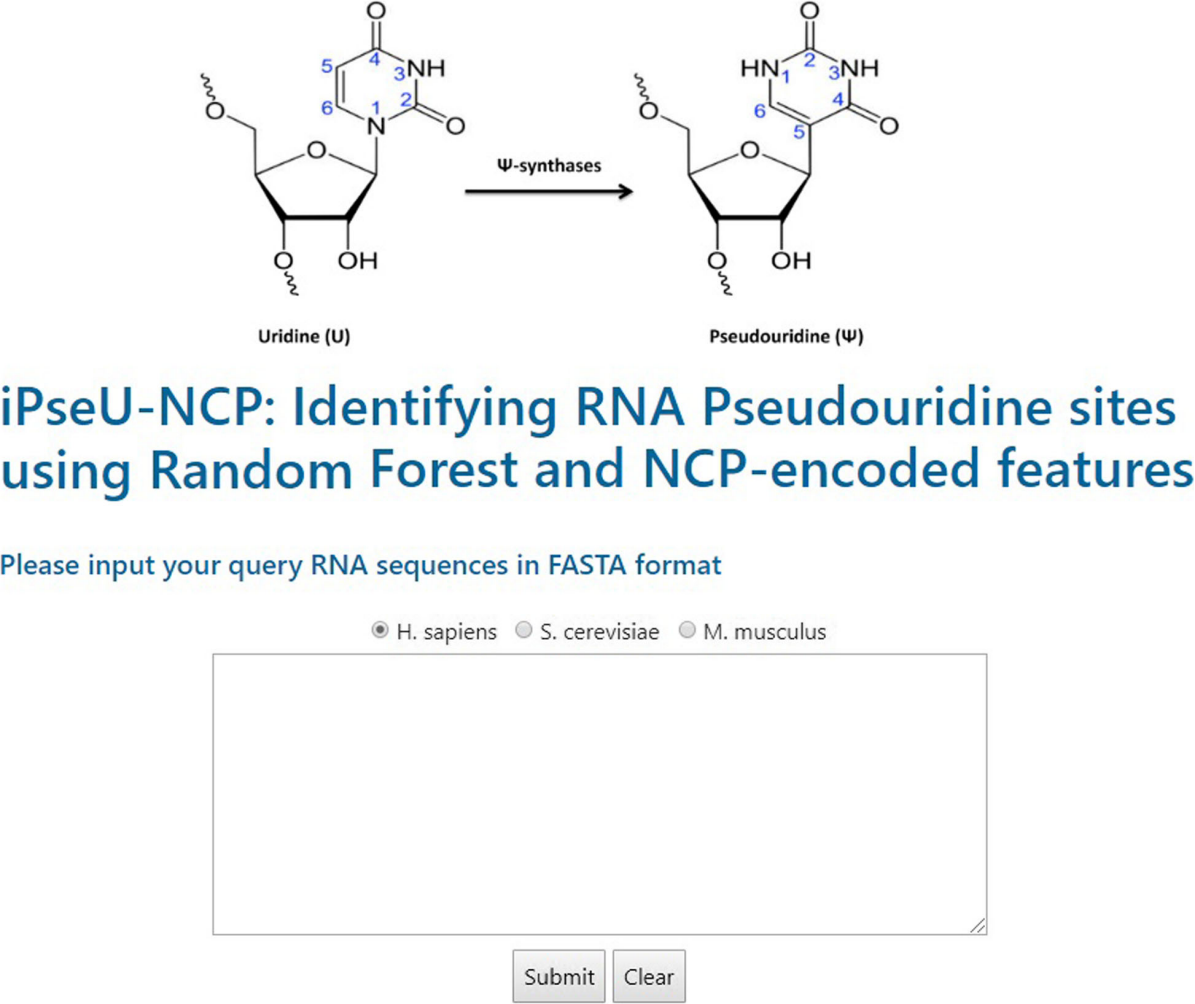
A snapshot of the iPseU-NCP web-server

To support experimental scientists to identify RPS, we developed an online publicly web server for iPseU-NCP at https://github.com/ngphubinh/iPseU-NCP with a user-friendly interface (Fig. 5). Users can use iPseU-NCP to identify RPS in an RNA sequence without consideration in dealing with mathematical details. At first, users fill the query box with an RNA sequence in the FASTA format along with one of three options corresponding to three species *H. sapiens*, *S. cerevisiae*, and *M. musculus*. Secondly, for each ‘U’ in the RNA sequence, a sliding window is placed given that ‘U’ is located in the central position of the window. The length of the window is 21, 31, and 21 for the three species: H, M, and S, respectively. This step generates several U-central fragments which are then converted into NPC-encoded features. Then the feature set of each U-central fragment is submitted to our iPseU-NCP model for identifying RPS. After the server finishes processing, the input RNA sequence is showed with all possible RPS which are displayed in red color.

## Conclusion

In this study, we proposed iPseU-NCP – an efficient computational framework combining the RF and NPC-encoded features to identify RPS. iPseU-NCP has significantly better performance than other state-of-the-art methods. In particular, iPseu-NPC can robustly and effectively address biological classification problems as evidenced by significant increases in most of the evaluation metrics, especially for accuracy and MCC. Our proposed method also shows a better-fitted application to address molecular genetics issues related to human beings as shown in the experimental results for all the development datasets and the independent test datasets.

## Methods

### Benchmark Dataset

The benchmark dataset comprises of three different development (or model training) sets and two different independent test sets corresponding to three species including *S. cerevisiae* (denoted as S), *M. musculus* (denoted as M), and *H. sapiens* (denoted as H). This dataset was collected from Chen et al.’s study [14]. S_628, M_944, and H_990 are the three development sets with 628, 944, and 990 samples, respecitvely. S_200 and H_200 are the two independent test sets with 200 samples for each set. In H_990 and M_944, each RNA sample has 21 nucleotides while in S_628, each RNA sample has 31 nucleotides. Positive and negative samples are both specified with a uridine located at the central position of the sequence. However, the central uridines of the positive samples are confirmed with feasibility of being pseudouridylated while those of the negative samples are confirmed with infeasibility of being pseudouridylated (Table 4).

**Table 4:** Data distribution of the training sets and the independent test sets

Dataset	Number of samples			Species	Group
	Possitive	Negative	Total		
S_628	314	314	628	*S. cerevisiae*	Training (Development)
M_944	472	472	944	*M. musculus*	
H_990	495	495	990	*H. sapiens*	
S_200	100	100	200	*S. cerevisiae*	Independent Test
H_200	100	100	200	*H. sapiens*	

### Overview of the Method

Figure 1 summarizes the steps involved in our study. For each of the three development sets (H_990, S_628, and M_944), we built a RF model using the NCP features extracted from RNA sequences stored in FASTA files. The optimal hyper-parameters of each model were determined through an exhaustive search over a specified grid of parameter values for the RF classifier using 5-fold cross-validation. The model performance corresponding to the optimal hyper-parameters was recorded as the 5-fold cross-validation performance on the development set. The model was retrained using the best hyper-parameters and then was tested with an independent test set if possible (H_200 or S_200) to for comparison with other existing state-of-the-art methods.

### NCP-encoding Scheme

The nucleotide chemical property (NCP) was used as the encoding scheme to convert each sequence sample into a 3×*n*-dimensional vector where *n* is the sequence length. An RNA sequence is formed of four different types of nucleotides, including adenine (A), guanine (G), cytosine (C), and uracil (U), which have distinct chemical structures and bonding. Guanine and adenine belong to the purine group with double fused aromatic rings while cytosine and uracil belong to the pyrimidine group with a single aromatic ring only. Both groups have their aromatic cyclic structures connecting to a sugar molecule. Besides, adenine and cytosine share the amino group as opposed to guanine and uracil with the keto group. On the other hand, the number of hydrogen bonds formed between adenine and uracil is smaller than that between guanine and cytosine. Therefore, the classification standard for these four kinds of nucleotides with three different groups of chemical properties has been set in a binary manner. A, C, G, and U are expressed by the combined coordinates as [1, 1, 1], [0, 1, 0], [1, 0, 0], and [0, 0, 1], respectively, based on the chemical properties (Table 5).

**Table 5:** NCP-encoding scheme

**Chemical property**	**Class**	**Binary Class**	**Nucleotides**
Cyclic Structure	Purine	1	A, G
	Pyrimidine	0	C, U
Functional Group	Amino	1	A, C
	Keto	0	G, U
Hydrogen Bond	Weak	1	A, U
	Strong	0	C, G

### Random Forest Classifier

The Random Forest (RF) algorithm [22] is an ensemble learning method that combines the “bagging" idea [23] and random selection of features [24] to construct multiple decision trees at training time, where the trees are slightly different from each other, and use the mode of the classes or the mean prediction of the individual trees as the output of a classification or a regression problem, respectively. Random forests address the drawback of decision trees: they tend to overfit the training data. We used the RF algorithm to train our predictive models using the NCP features extracted from RNA sequences in the development sets and then tested the models on the independent test sets. The number of trees in each forest was fixed at 200, and there were two hyper-parameters which were determined from an exhaustive search over a specified grid of parameter values using 5-fold cross-validation on each development set. They were the maximum depth of the tree (max_depth) and the number of features (max_features) to consider when looking for the best split at each node of the tree. For each dataset, we searched for the best max_depth from the range 2 to 6, and the best max_features were determined from 20% to 80%, with step size of 5%, of the total number of features, i.e., the length of each NCP vector.

### Model Evaluation

To assess the model performance, several standard metrics comprising of Matthews’s correlation coefficient (MCC), Accuracy (ACC), Specificity (SP), and Sensitivity (SN) were adopted. TP, FP, TN, and FN stand for True Positive, False Positive, True Negative, and False Negative values, respectively. The mathematical formulas of these evaluation metrics are expressed below.

1 $$ Sensitivity \,(SN) = \frac{{TP}}{{TP + FN}}  $$

2 $$ Specificity \,(SP) = \frac{{TN}}{{TN + FP}}  $$

3 $$ Accuracy \,(ACC) = \frac{{TP+TN}}{{TP + TN + FP + FN}}  $$

4 $$ MCC \,=\, \frac{{TP \times TN - FP \times FN}}{{\sqrt {(TP \,+\, FP)(TP + FN)(TN + FP)(TN + FN)} }}  $$

## Data Availability

The benchmark dataset used in this study were collected from the previous work of Chen et al., 2016. The benchmark dataset were downloaded from the Supplementary section of the paper entitled “iRNA-PseU: Identifying RNA pseudouridine sites” of Chen et al.. A web server implementing the proposed method is available at https://github.com/ngphubinh/iPseU-NCP.

## Abbreviations

AUC: Area under the ROC curve
DC: Dinucleotide composition
DNA: Deoxyribonucleic acid
MCC: Matthew’s correlation coefficient
NC: Nucleotide composition
NCP: Nucleotide chemical properties
PSDP: Position-specific dinucleotide propensity
PseDNC: Pseudo dinucleotide composition
PSNP: Position-specific nucleotide propensity
RF: Random forest
RNA: Ribonucleic acid
ROC: Reciever operating characteristic
RPS: RNA pseudouridine site
SVM: Support vector machine
